# Optimized measurement methods and systems for the dielectric properties of active biological tissues in the 10Hz-100 MHz frequency range

**DOI:** 10.3389/fphys.2025.1537537

**Published:** 2025-01-30

**Authors:** Yueying Shi, Xiaoxiao Bai, Jingrong Yang, Xinyu Wu, Lei Wang

**Affiliations:** ^1^ School of Stomatology, Xi’an Medical University, Xi’an, China; ^2^ Institute of Medical Research, Northwestern Polytechnical University, Xi’an, China

**Keywords:** dielectric properties, active biological tissue, 10Hz-100 MHz frequency range, dualpurpose measuring cell, electrode polarization, distributed parameters, measurement correction

## Abstract

The dielectric properties of active biological tissues within the 10Hz-100 MHz frequency range contain rich information about tissue morphology and function. Accurately understanding the dielectric properties of active human tissues holds significant value for disease diagnosis and electromagnetic protection. However, accurately measuring these properties has been challenging due to factors such as electrode polarization and distribution parameters. This study has developed a dual-purpose measuring cell that supports both four-electrode and two-electrode impedance measurements. Leveraging this development, we have established a system and methodology that is well-suited for the dielectric property measurement of active biological tissues within the frequency range of 10Hz to 100 MHz. Our measurements of dielectric properties in NaCl solutions of varying concentrations and pig liver tissues demonstrate the system’s high accuracy and repeatability. For NaCl solutions, the maximum relative deviation is only 6.34%, with an average deviation of less than 1.5%. For pig liver tissues, the overall relative deviation is below 6%. Through the integration of the four-electrode and two-electrode measurement systems, we have successfully addressed the challenges of electrode polarization at low frequencies and the influence of distribution parameters at high frequencies, achieving a significant improvement in measurement accuracy across the spectrum.

## 1 Introduction

The dielectric properties of biological tissues are the characteristics of how the tissues respond to an externally applied electromagnetic field and are intrinsic to the tissues when subjected to such fields (Wang et al., 2021). Conductivity and permittivity are the crucial parameters among these characteristics.

Since Hermann first discovered the electrical conduction properties of skeletal muscle in 1871, research on the dielectric properties of biological tissues has spanned over 140 years. During this period, significant advancements were made, such as the Cole-Cole model for the dielectric properties of biological tissues introduced by the Cole brothers (Cole and Cole, 1941), and Schwan’s understanding of the α , β , and γ dispersions in the dielectric properties of biological tissues (Schwan, 1957). These studies greatly enhanced our understanding of the manifestations and mechanisms underlying the dielectric properties of biological tissues.

At present, the consensus among scholars is that the dielectric properties of biological tissues can be categorized into four distinct dispersion regions, α , β , γ and δ dispersions (Foster and Schwan, 1989; Braun, Schmollngruber, and Steinhauser, 2017; Liu et al., 2024). The α dispersion region (10 Hz ∼ 10^3^ Hz), where the dielectric properties are closely related to the diffusion of ions. In β dispersion region (10^3^ Hz ∼ 10^7^ Hz), the dielectric properties are primarily associated with the capacitive effect of cell membranes, especially the Maxwell-Wagner effect caused by polarization at the membrane surface. The γ dispersion region (10^7^ Hz ∼ 10^10^ Hz) in which the dielectric properties are mainly caused by the dipole motion of large molecules and organelles within cells. In δ dispersion region (>10^10^ Hz), the dielectric properties are mainly caused by the dipole motion of water molecules and other large molecular substances.

Hence, it is evident that the dielectric properties of biological tissues are intricately tied to the tissues’ morphology, structure, composition, and functional state. Some studies have found that the conductivity of liver cancer tissue is more than 20% higher than that of healthy tissue, primarily due to the increased water content in cancer cells, which has a higher conductivity (Wang et al., 2014; Prakash et al., 2015). Other studies have shown that ischemia can lead to an increase in brain tissue impedance by over 15%, closely related to the reduction of blood and cell swelling (Harting et al., 2010; Song et al., 2018). Understanding these relationships is crucial for advancing our knowledge of biological processes and developing medical applications that leverage these properties.

From the mechanisms discussed, it is clear that the dielectric properties within the frequency band below 100 MHz are highly related to the morphological and structural information at both the tissue and cellular level. Accurately understanding the dielectric properties in this frequency range is crucial for variety of applications, including disease diagnosis (Awal et al., 2024), electromagnetic protection (Hamedani et al., 2022), electrical impedance imaging (Kaatze, 2013; Lee et al., 2021), and disease treatment (Gaynor, Hagberg, and Gurfein, 2018). However, although the dielectric properties of biological tissues in the higher frequency range, particularly in the microwave frequency range show a high degree of consistency in their results (Gabriel and Peyman, 2006; Peyman et al., 2007; Peyman, 2011; Naqvi et al., 2021), the dielectric properties in the lower frequency range ( α and β dispersion region) continue to be a focus of ongoing research and debate.

In addition to the source and nature of the samples, a key factor contributing to this outcome is the measurement method. The selection of an appropriate measurement technique is essential for achieving consistent results. Techniques such as the open-ended coaxial probe method (La Gioia et al., 2018), the Transmission/Reflection line method (Smith et al., 2024), and the Resonant Technique (Choi et al., 2022) each have their limitations, especially at lower frequencies, where factors like electrode polarization and tissue impedance can introduce considerable variability in the measurements (Shetty et al., 2020).

Impedance measurement method is a technique used to measure the impedance of biological tissues within the mid-low frequency band, mainly employing two-electrode and four-electrode method. Each of these methods comes with its own set of advantages and limitations. The two-electrode method utilizes the same pair of electrodes for both current application and voltage measurement. This can lead to inaccuracies, particularly in the α dispersion region, due to the influence of contact impedance and the electrode resistance on the measurement results (Dai, Xue, and Zhang, 2014; Lee et al., 2022). The four-electrode method uses two separate pairs of electrodes-one for applying a known current and the other for voltage measurement. This approach helps to minimize the contact impedance and electrode resistance. However, distributed parameters include inductance and capacitance can affect the performance of the measurement circuit, especially in the radio frequency band ( β dispersion region) (Pradhan et al., 2021; Sekine, 2020).

In summary, electrode polarization and distributed parameter issues are key factors affecting the dielectric property measurements of biological tissues in the α and β dispersion regions. A challenge in this field is how to suppress the electrode polarization effects in the low-frequency band and control the distributed parameter effects in the radio frequency band while maintaining tissue viability, in order to accurately measure the dielectric properties of tissues.

This study introduces a novel measurement method that integrates the four-electrode and two-electrode approaches to measure the dielectric properties of biological tissues in the frequency range of 10 Hz–100 MHz. A two/four-electrode dual-purpose measuring cell has been developed to enable rapid switching between the four-electrode and two-electrode methods. The four-electrode method was used to measure the impedance below 1 MHz to eliminate the polarization effects of the electrodes and contact resistance. The two-electrode method was used to measure the impedance above 1 MHz to eliminate the effect of stray capacitance and inductance.

By combining these two methods, this novel measurement technique adeptly tackles the challenges posed by electrode polarization and distributed parameters. It ensures uniformity in measurement conditions, which is paramount for enhancing the precision of dielectric property measurements. Moreover, this approach provides a more nuanced and detailed view of the dielectric behavior of biological tissues across a wide range of frequencies. It allows researchers to capture the complex interactions between electrical fields and biological matter with greater accuracy, which is crucial for applications in medicine, biotechnology, and other fields where understanding tissue dielectric properties is key.

## 2 Materials and methods

### 2.1 Two/four-electrode dual-purpose measuring cell

**FIGURE 1:**
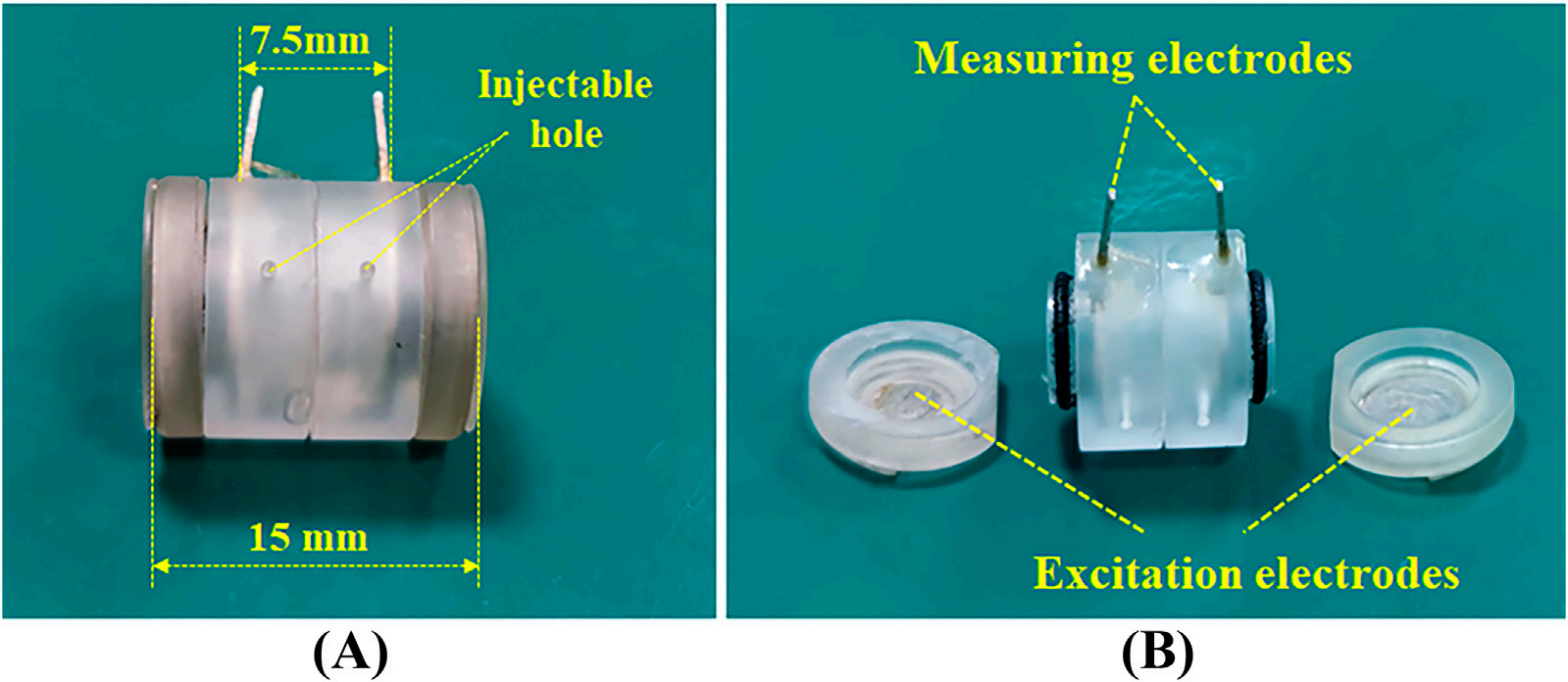
Two/four-electrode dual-purpose measuring cell.

We have designed a cylindrical dielectric property measuring cell tailored for biological soft tissues, as shown in Figure 1, to meet the requirements of both two-electrode and four-electrode methods. The measuring cell is made of transparent organic glass, with the inner diameter is 5mm, the outer diameter is 8mm, and the length is 15 mm. At both ends, two circular silver electrodes with a thickness of 0.4 mm are fixed to the organic glass box cover and can be conveniently removed with the cover for filling the tissue to be measured or for cleaning.

An O-ring made of rubber is placed between the box cover and the box body to prevent leakage. Two annular silver electrodes with a thickness of 0.4 mm are embedded in the middle section of the box, with the inner side of the rings maintaining the same cylindrical surface as the inner cavity of the box. The two annular electrodes are spaced 7.5 mm apart. The box body features two round holes with a diameter of 1mm, which can be used for liquid injection and gas release.

During dielectric property measurements, the excitation signal is injected through the two circular electrodes at the ends, and the response signal can be directly measured from these two electrodes (two-electrode method), or it can be measured through the intermediate annular electrodes embedded in the box (four-electrode method).

Before measuring, it is necessary to immerse the electrodes in 75% medical alcohol for more than 10 min to perform sterilization. Then, soak them in saline solution for more than 30 min to maintain a relatively stable polarization state of the electrodes. This procedure ensures that the electrodes are clean and free from contaminants that could affect the accuracy of the measurements, and it helps to stabilize the electrode polarization, which is crucial for obtaining consistent and reliable results in dielectric property measurements.

### 2.2 Four-electrode system for dielectric property measurement

**FIGURE 2:**
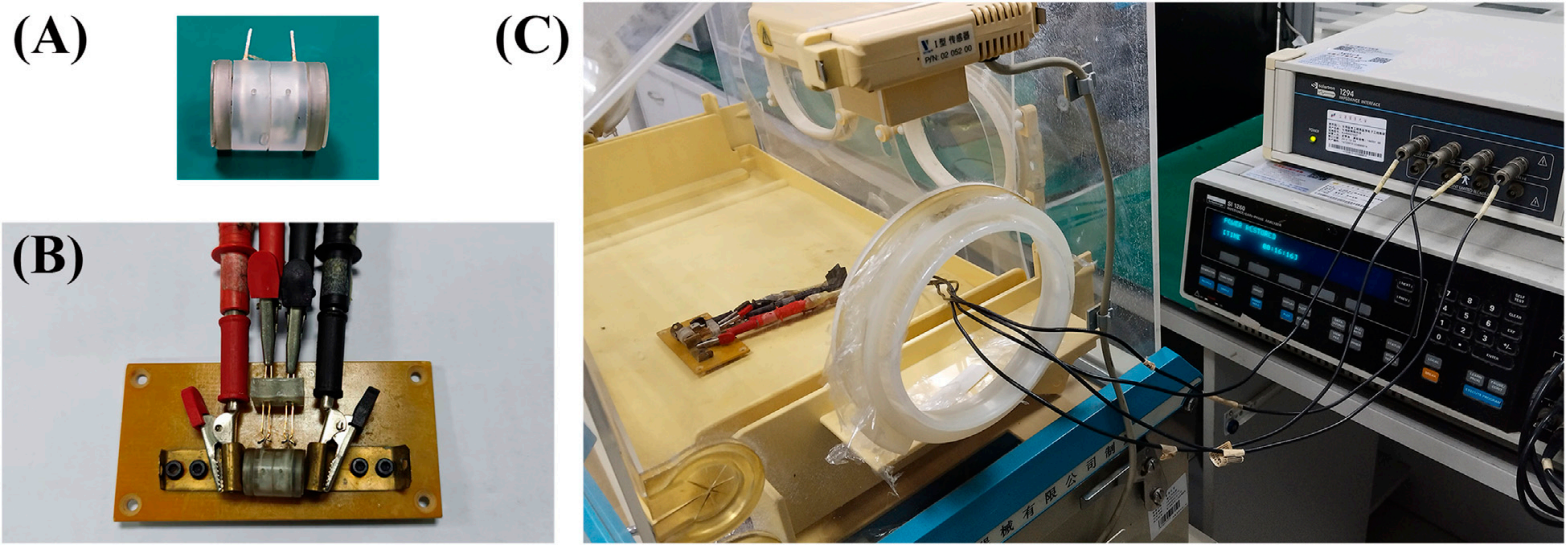
Four-electrode system for dielectric property measurement. **(A)** Measuring cell. **(B)** The measuring cell is placed on a measuring board which serves as the bridge connecting the measuring cell and the impedance measurement system. **(C)** The measuring board and the measuring cell are placed inside an infant incubator and connected to the impedance measurement system.

The four-electrode measurement system presented in Figure 2, comprises a Solartron 1260A frequency response analyzer and a Solartron 1,294 impedance measurement interface (Schlumberger, United Kingdom). The excitation terminals of the Solartron 1,294 are connected to the circular electrodes at both ends of the measuring cell, while the measurement terminals are connected to the annular electrodes in the middle section of the cell (Figure 2B). The entire process of sample installation and measurement is conducted in an infant incubator to maintain a stable temperature (37°C) and humidity (90%) environment within 30 min.

The system is connected to the main control computer via USB-GPIB, and measurement methods and parameter settings are selected and configured using the Zplot measurement software. This software allows for the measurement of the real part, imaginary part, modulus, and phase of the complex impedance of the target under test. Additionally, these parameters can be mathematically converted into equivalent capacitance and admittance of the target and saved to the computer.

Due to the significant impact of the distributed parameters of electrode leads at high frequencies, this system is primarily used for dielectric property measurements in the frequency range of 1Hz to 1 MHz. Since the Solartron 1,294 impedance measurement interface supports true differential four-terminal connections, it has high input impedance and sensitivity to both voltage and current. Therefore, this system can more effectively suppress the influence of high electrode impedance caused by electrode polarization, achieving high-precision measurements in the low frequencies.

### 2.3 Two-electrode system for dielectric property measurement

**FIGURE 3:**
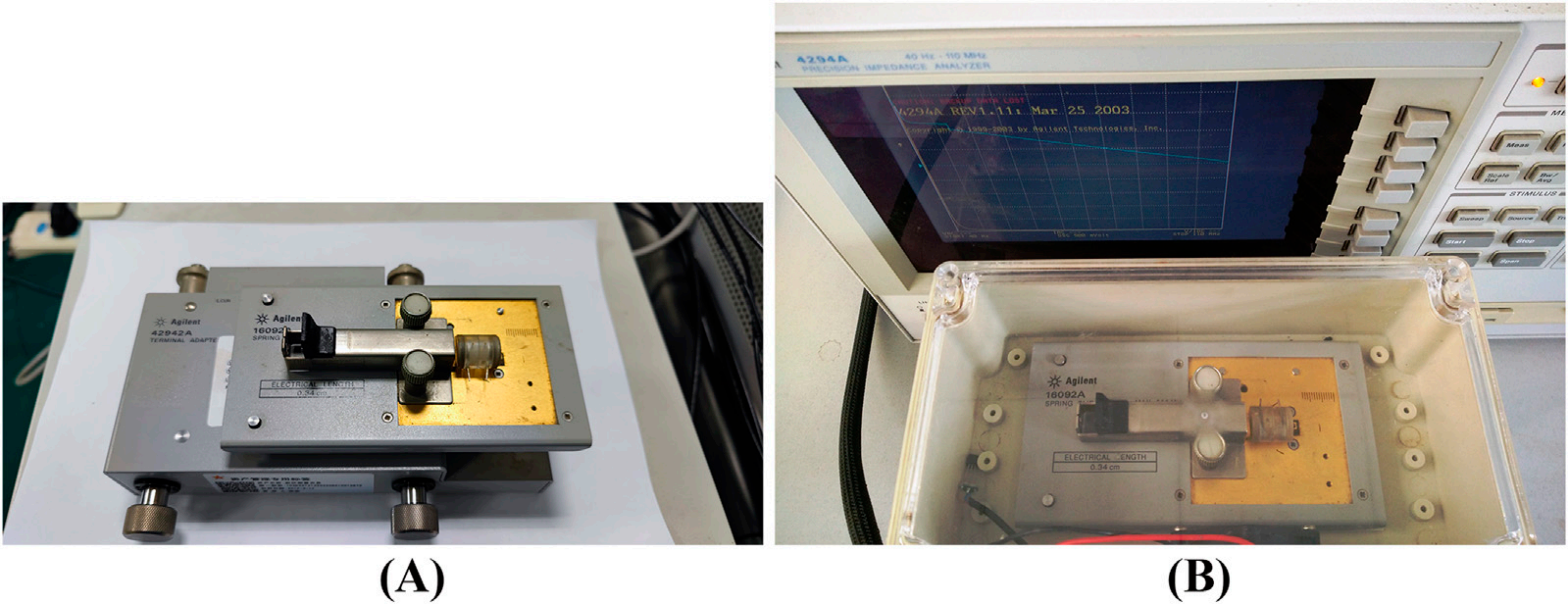
Tour-electrode system for dielectric property measurement. **(A)** The Agilent 16092A clamp along with the measuring cell is connected to the Agilent 42942A Terminal Adapter. **(B)** The measuring cell placed in a temperature-controlled incubator is connected to the Agilent 4294A Impedance Analyzer.

As shown in Figure 3, the two-electrode measurement system is primarily based on the Agilent 4294A Impedance Analyzer (Agilent Technologies, United States). The Agilent 42942A Terminal Adapter is used to transform the four-terminal configuration of the impedance analyzer’s measurement ends into a 7 mm two-terminal configuration (Figure 3A). The Agilent 16092A clamp serves as the interface to connect the circular electrodes at both ends of the dual-purpose measuring cell to the measurement system. The Agilent 16092A clamp, along with the measuring cell, is installed together in a temperature-controlled incubator to maintain the sample temperature (37°C). The system is connected to the main control computer via GBIP, and Intuilink software is integrated within Office software, allowing measurement data to be imported into Excel for analysis (Figure 3B).

The two-electrode measurement system is used to measure the real and imaginary parts of the impedance of biological tissues in the frequency range of 40Hz to 100 MHz. Then mathematically convert them into equivalent conductance and capacitance. Before measurement, Agilent 42942A and Agilent 16092A are calibrated with standard calibration terminals provided by the manufacturer to ensure accuracy and reliability of the data obtained.

### 2.4 Measurement error correction and dielectric parameter extraction

In dielectric property measurements, there are some sources of systematic error, including the dimensions of the measuring cell, the nonlinearity of the measuring instruments, and the distributed parameters of electrodes and connecting wires. To improve the accuracy of the measurements, these errors need to be corrected. Corrections for both four-electrode and two-electrode methods are based on the 12.88 m/cm (25°C) standard conductivity solution produced by Mettler Toledo.

#### 2.4.1 Four-electrode method measurement correction

Recognizing that the impact of distributed parameters is less significant in the low-frequency band, the systematic errors are primarily due to deviations in the dimensions of the measuring cell and the nonlinearity of the measuring systems.

Therefore, following the method of Gabriel et al. (Gabriel, Peyman, and Grant, 2009), we use the ratio of the nominal value to the measured value of a standard conductivity solution to determine the measuring cell constant ( K ), and correct the measurement results accordingly.

$$\sigma_{meas}\left(\omega \right)=\sigma_{raw}\left(\omega \right)K\left(\omega \right)$$

$$\varepsilon_{real}\left(\omega \right)=\frac{C\cdot K\left(\omega \right)}{\varepsilon_{meas}\left(\omega \right)}=\frac{-Im\cdot K\left(\omega \right)}{\omega \left({Re}^{2}+{Im}^{2}\right)\cdot \varepsilon_{meas}\left(\omega \right)}$$

Where, $\sigma_{\text{meas}}$ and $\sigma_{\text{raw}}$ are the measured conductivity and standard conductivity, respectively. $\varepsilon_{\text{real}}$ is the corrected value of measured permittivity $\varepsilon_{\text{raw}}$ . The coefficient $C=\frac{-Im}{\omega \left(Re^{2}+Im^{2}\right)}$ is the equivalent capacitance of the tested object which can be obtained through the impedance measurement instrument or through the calculation using an equivalent circuit model. Re and Im are the real part and imaginary part of measured impedance at angle frequency ω .

#### 2.4.2 Two-electrode method measurement correction

**FIGURE 4:**
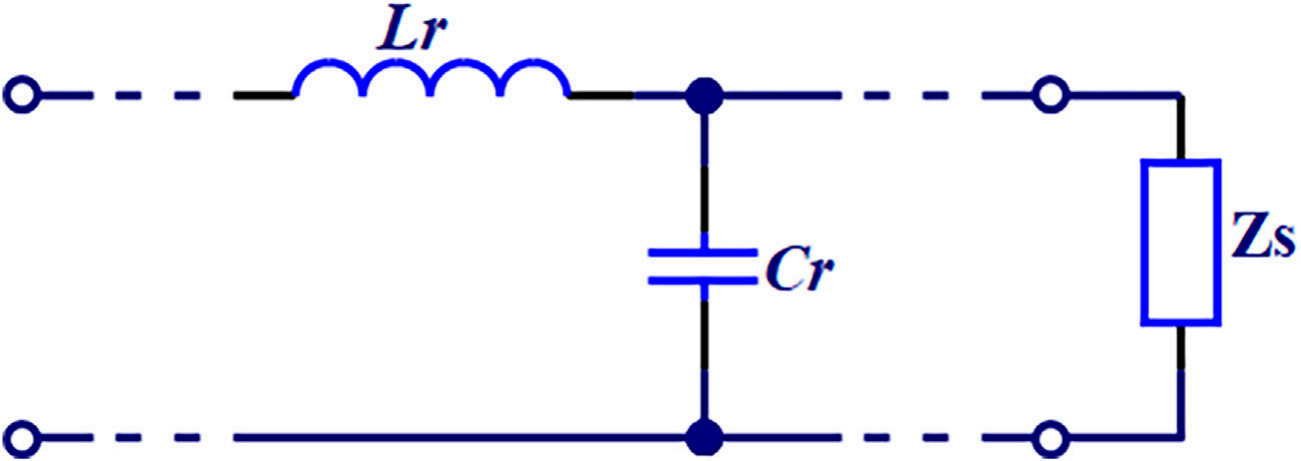
Distributed parameter equivalent circuit.

The two-electrode method developed in this study has reduced the stray capacitance caused by electrode leads using specialized adapters and clamps. Moreover, the impact of distributed parameters has been further minimized through internal calibration of the instrument before measurement. However, in actual measurement, stray capacitance caused by the leakage of electrical lines from wires and the electrodes at both ends of the measuring cell, as well as the residual inductance of the connecting wires, still impact measurements in high-frequency range. Therefore, based on the equivalent circuit model shown in Figure 4, we have developed the following correction method.

$$C_{s}=\frac{C_{i}\left(1+\omega^{2}L_{r}C_{i}\right)+L_{r}{G_{i}}^{2}}{{\left(1+\omega^{2}L_{r}C_{i}\right)}^{2}+{\left(\omega L_{r}G_{i}\right)}^{2}}-C_{r}$$

$$G_{s}=\frac{G_{i}}{{\left(1+\omega^{2}L_{r}C_{i}\right)}^{2}+{\left(\omega L_{r}G_{i}\right)}^{2}}$$

Where, $C_{s}$ and $G_{s}$ are the equivalent parallel capacitance and conductance, respectively. $C_{i}$ and $G_{i}$ represent the measured capacitance and conductance, respectively. $C_{r}$ and $L_{r}$ are the stray capacitance and residual inductance, respectively. Stray capacitance $C_{r}$ can be calculated using measurements from two or more known dielectric materials (in this study, distilled water and air are used) as follows:

$$C_{r}=C_{air}-\frac{C_{H_{2}O}-C_{air}}{\varepsilon_{H_{2}O}-\varepsilon_{air}}$$

Where, $C_{air}$ and $C_{H_{2}O}$ are the equivalent capacitance between the two excitation electrodes of the measuring cell when the box is filled with air and distilled water, respectively. $\varepsilon_{H_{2}O}$ represents the relative permittivity of air at 37°C is approximately 1, and $\varepsilon_{air}$ represents the relative permittivity of distilled water is approximately 78.36. Residual inductance $L_{r}$ can be calculated by the following equation:

$$L_{r}=\frac{C_{s}+C_{r}-C_{i0}}{{G_{i0}}^{2}}$$

Where, $C_{i0}$ and $G_{i0}$ are the measured capacitance and conductance of a standard conductivity solution at low frequencies.

### 2.5 Experimental method

#### 2.5.1 Dielectric property measurement of NaCl

At 37°C, NaCl solutions with concentrations of 0.01, 0.03, 0.05, and 0.07 Mol/L were prepared using distilled water and analytical-grade NaCl. Each solution was prepared to a volume of 500 mL. A portion of each solution was used to thoroughly rinse the measuring cell and electrodes, followed by an immersion of the cell and electrodes for over 30 min to stabilize. Thereafter, the measuring cell was filled with a NaCl solution of the same concentration as the rinsing and soaking solutions. The measurement results were corrected based on the calibration parameters.

#### 2.5.2 Dielectric property measurement of porcine liver

At 37°C, the measuring cell is thoroughly rinsed and soaked in physiological saline for over 30 min. The experimental subject is a 10 kg (45 days old) pig, sourced from the Laboratory Animal Center of the Fourth Military Medical University, China. All experiments were approved by the Ethics Committee of the Fourth Military Medical University (approval no. FMMU-E-III-001 (1), 2007).

Following routine anesthesia, the pig is securely positioned supine on the dissection table. Under sterile conditions, the abdominal cavity is opened along the midline below the xiphoid process to expose the liver. Vessels are clamped with hemostatic forceps in the direction of the porta to block blood supply. Subsequently, the left lobe of the liver is meticulously excised. Six adjacent samples are then cut along the same direction, trimmed and moistened with physiological saline.

The length of these samples is 15mm, which is consistent with the length of the measuring cell. The width and height are both 5mm, which are consistent with the inner diameter of the measuring cell. This tailored fit not only facilitates the quick and easy loading of samples into the measurement cell but also ensures that the samples fill the cell completely without any gaps, thereby minimizing measurement errors. Then, all the samples were filled into six measuring cells for measurement. All operations are controlled within 30 min starting from the clamping of the blood supply to reduce tissue degradation and ensure the freshness and viability of the samples. Finally, the measured results were corrected based on the calibration parameters.

## 3 Results

### 3.1 Optimal frequency range of the four-electrode and the two-electrode methods

The dielectric properties of the measured material were obtained using the four-electrode method for the frequency range of 10Hz to 1 MHz and the two-electrode method for the frequency range of 40Hz to 100 MHz. To verify the accuracy of the results and the valid frequency range of the measured data, we analyzed the conductivity of NaCl solutions at 0.01 Mol/L and 0.05 Mol/L, as well as the conductivity and permittivity of the porcine liver tissue.

**FIGURE 5:**
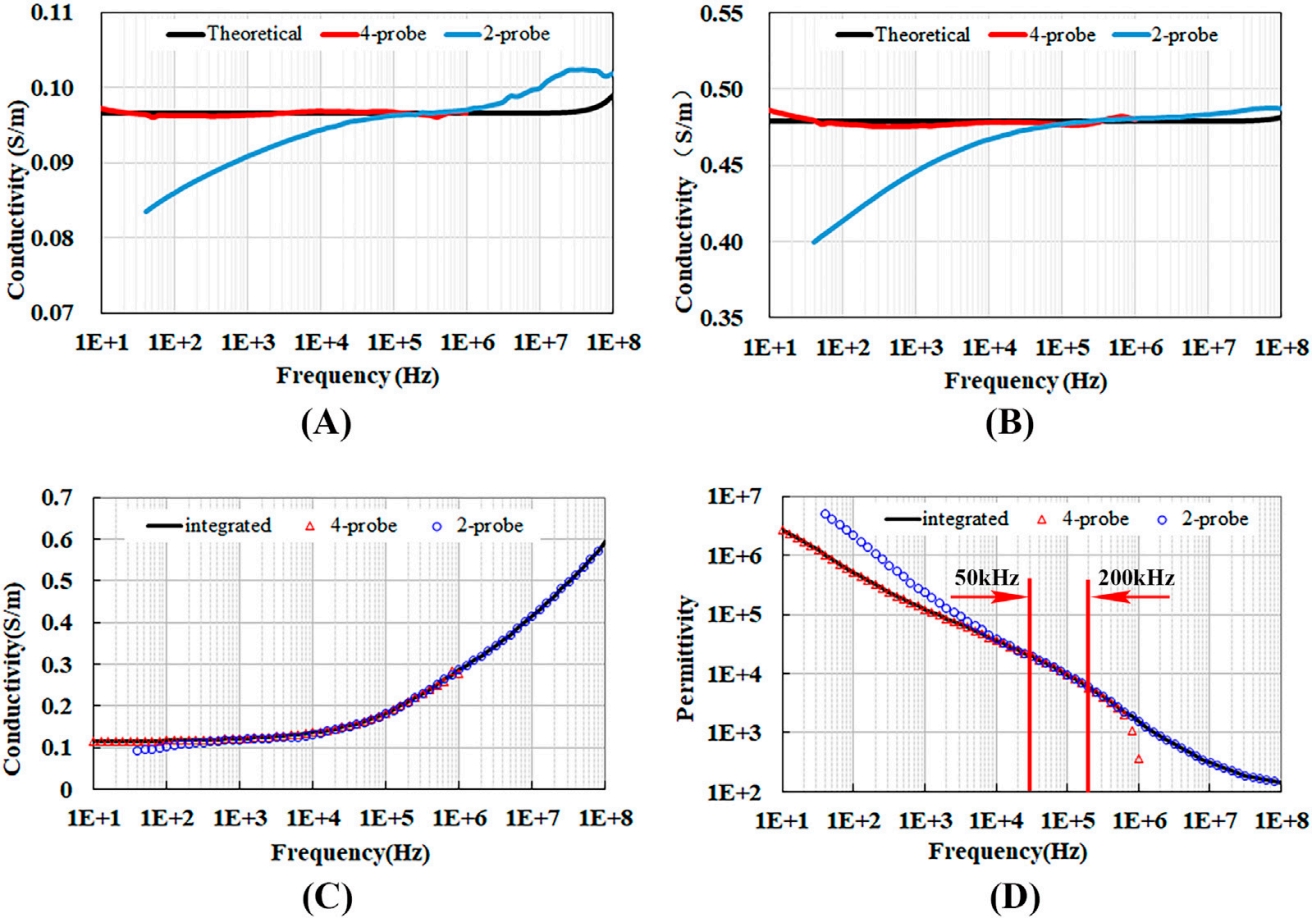
Conductivity and permittivity measured using the four-electrode and two-electrode methods. **(A)** Conductivity of 0.01 Mol/L NaCl solution, **(B)** Conductivity of 0.05 Mol/L NaCl solution, **(C)** Conductivity of porcine liver tissue, **(D)** Permittivity of porcine liver tissue.

Figures 5A, B present the measurement results and theoretical calculations for the conductivity of NaCl solutions. It can be observed that the conductivity measured via the four-electrode method below 1 MHz closely aligns with theoretical values. The conductivity measured via the two-electrode method above 10 kHz shows close agreement with theoretical values, while significant deviations are observed below 10 kHz, with the deviation increasing rapidly as the frequency decreases. This suggests that the measurements using two-electrode method in this frequency range are greatly affected by electrode polarization, leading to poor credibility of the results.

Figures 5C, D shows the measured conductivity and permittivity of porcine liver tissue. Below 4 kHz, due to the influence of contact impedance, the conductivity measured by the two-electrode method is lower than that measured by the four-electrode method. While the permittivity measured by the two-electrode method exceeds that obtained by the four-electrode method, with the difference widening as the frequency decreases. Above 400 kHz, the conductivity and permittivity measured by the four-electrode method begin to deviate from those obtained by the two-electrode method due to the impact of distributed parameters. Between 4 kHz and 400 kHz, and particularly within the frequency range of 50 kHz–200 kHz, there is a complete concordance between the results from both measurement methods.

Given the insights from our study, we have established the following guidelines for determining the dielectric properties of the target in our future research. For frequencies below 50 kHz, we will rely on the measurement results from the four-electrode method. This decision is informed by the pronounced effects of contact impedance at these lower frequencies, which can significantly bias the results from the two-electrode method, leading to lower conductivity and higher permittivity.

For frequencies ranging from 50 kHz to 200 kHz, we will utilize a weighted average of the measurements from both the four-electrode and two-electrode methods. This approach is justified by the fact that, within this frequency band, both methods provide consistent results. By using a weighted average, we can leverage the combined precision and reliability of both techniques, thereby enhancing the overall accuracy of our dielectric property determinations.

For frequencies above 200 kHz, we will rely on the measurement results from the two-electrode method. This decision stems from the observation that the four-electrode method’s measurements begin to deviate from those of the two-electrode method at these higher frequencies. The deviation is likely attributed to the effects of distributed parameters, which can impair the accuracy of the four-electrode method’s readings.

### 3.2 Measurement results of NaCl solution

**FIGURE 6:**
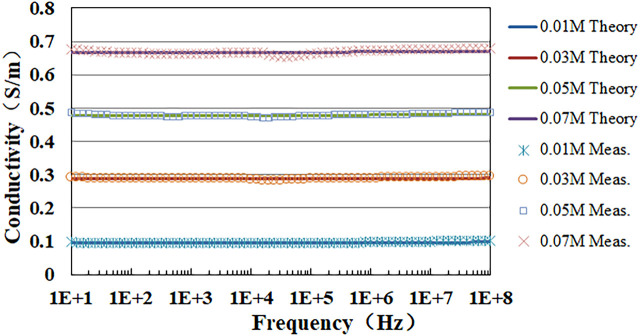
Theoretical and measured conductivity values of NaCl solutions with different concentrations.

Following the established protocols, we have determined the conductivities of NaCl solutions with concentrations of 0.01, 0.03, 0.05, and 0.07 Mol/L. Figure 6 depicts the frequency-dependent variation of both the measured and theoretical conductivity values for these four NaCl solutions. Notably, the comparison indicates a high degree of correspondence between the measured and theoretical values.

Furthermore, the average standard deviations for the four different concentrations were 1.76 × 10^−3^, 2.68 × 10^−3^, 4.66 × 10^−3^, and 5.95 × 10^−3^, representing 1.79%, 0.92%, 0.96%, and 0.88% of the mean values, respectively. Given that the measurement samples of the same concentration all came from the same solution and that sodium chloride solutions can maintain their chemical stability at 37°C, the average standard deviations were mainly attributed to the measurement system and method. This minimal variability suggested the high stability and accuracy of our measurement system and methods.

Relative error $E_{r}$ was used to evaluate the accuracy of our methods for determining tissue conductivity across different measurement frequency ranges. Relative error quantifies the discrepancy between measured values and theoretical values, providing insight into measurement accuracy.

$$E_{r}\left(f_{i}\right)=\left|\frac{\sigma_{m}\left(f_{i}\right)-\sigma_{t}\left(f_{i}\right)}{\sigma_{t}\left(f_{i}\right)}\right|\times 100\%$$

Where, $\sigma_{m}$ and $\sigma_{t}$ represent measured and theoretical conductivity at the measured frequency $f_{i}$ , respectively. The average and maximum value of $E_{r}$ are shown in Table 1. NaCl solution with concentrations of 0.01 Mol/L exhibits the highest average relative error $E_{rave}$ and maximum relative error $E_{r\;\max}$ , peaking at 1.46% and 6.34%. With an increase in the concentration of NaCl solutions, both $E_{rave}$ and $E_{r\;\max}$ progressively diminish, dropping to 0.47% and 1.27% when the solution concentration reaches 0.07 Mol/L. This might be attributed to the ions in the solution. At lower concentrations, fewer ions are available, resulting in a weaker signal and greater susceptibility to various interfering factors, which in turn leads to a larger $E_{r}$ . As the concentration increases, the ion count rises, enhancing the signal and consequently reducing the $E_{r}$ . The regression coefficients $R^{2}$ for the four concentrations of sodium chloride solution are all above 0.98, indicating an exceptionally strong linear relationship between the measured values and the theoretical values. This suggests that the experimental data is highly reliable and accurate.

**TABLE 1:** Average and maximum relative errors between measured and theoretical conductivity of NaCl solutions with different concentrations.

Concentration (Mol/L)	Average relative error $E_{rave}$ (%)	Maximum relative error $E_{r\;\max}$ (%)	Regression coefficient $R^{2}$
0.01	1.46	6.34	0.9876
0.03	0.85	3.01	0.9916
0.05	0.48	1.75	0.9971
0.07	0.47	1.27	0.9983

### 3.3 Measurement results of porcine liver tissue

**FIGURE 7:**
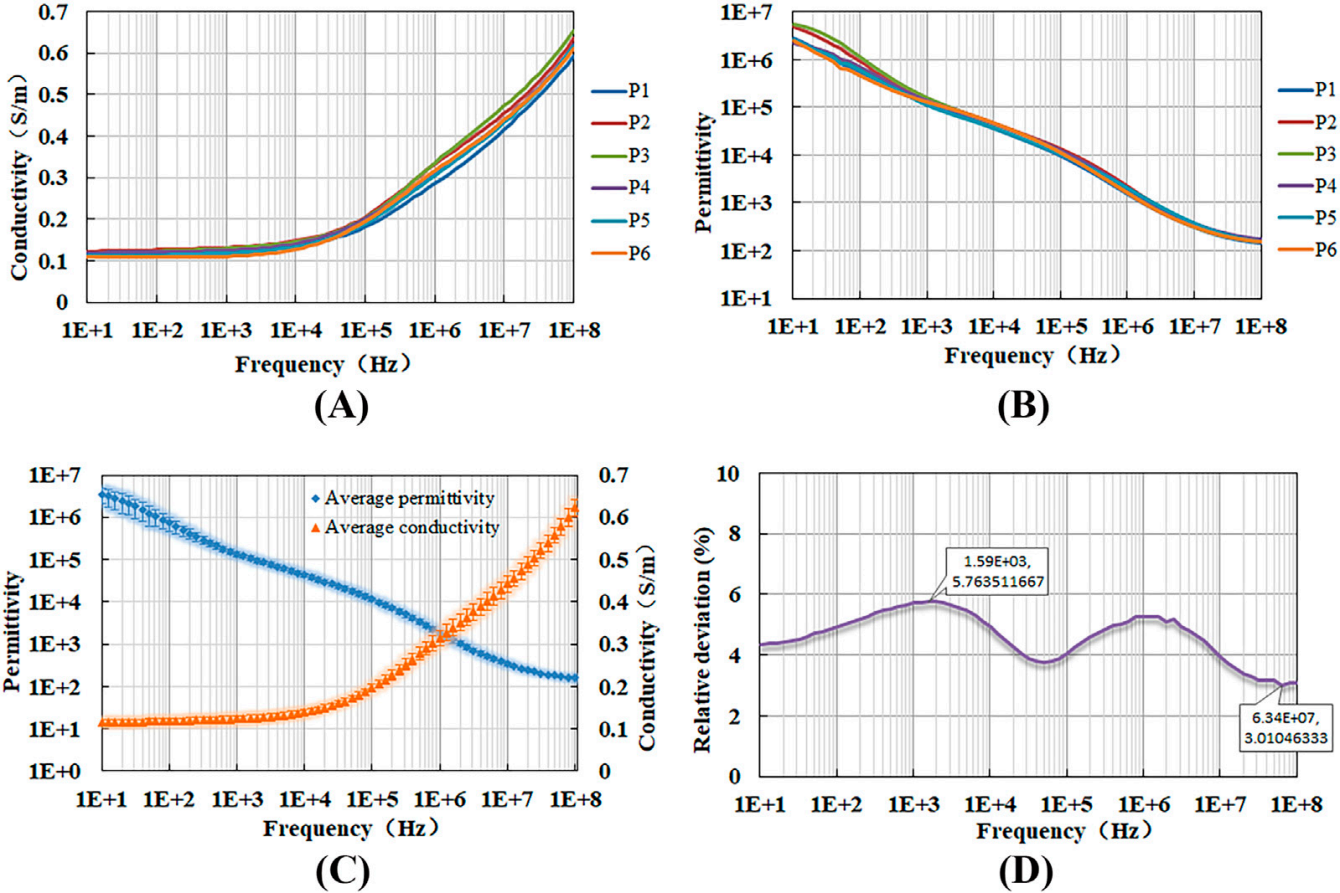
Variation of **(A)** conductivity, **(B)** permittivity, **(C)** the mean value and standard deviation for these properties, and **(D)** the coefficient of variation with measurement frequency for porcine liver tissues.

Figures 7A, B display the measured conductivity and permittivity of six porcine liver tissue samples, while Figure 7C summarizes the mean value and standard deviation for both conductivity and permittivity. Notably, the dielectric properties of these samples are found to be close to each other. To assess the consistency of these measurements, we calculated the relative deviation (standard deviation as a percentage of the mean) for conductivity across all six samples at each frequency (Figure 7D). The observed deviations, ranging from 3% to 6%, which is relatively small, indicating good repeatability of our measurement method. This consistency suggests that the method is reliable for accurately determining the dielectric properties of the tissue.

## 4 Discussion

The dielectric properties of biological tissues within the frequency range of 10 Hz–100 MHz are intricately linked to tissue types, functional states, and the morphological structure of cells and organelles. These properties are crucial for their significant potential in biomedical research and clinical practice. This study has developed a novel standardized method for measuring the dielectric properties of active biological tissues in the frequency range of 10 Hz–100 MHz. Our method is designed to significantly reduce the effects of electrode polarization and the influence of distributed parameters, thereby enhancing the accuracy and reliability of the measurement outcomes.

### 4.1 The advantage of the measuring cell

The measuring cell designed in this study is a novel approach that addresses specific challenges associated with *in vivo* tissue measurements. It is engineered to provide a controlled environment for tissue samples, ensuring that the electric field is distributed uniformly and predictably. This design not only minimizes the effects of tissue boundary changes and the influence of surrounding tissues on the electric field, but also prevents tissue from being subjected to undue pressure during the measurement process, thus avoiding deformation that can skew results.

What’s more important is that this versatile device can swiftly and flexibly switch between the two-electrode and four-electrode methods, accommodating the measurement requirements for dielectric characteristics across a broad spectrum of frequencies. It is beneficial for diminishing the impact of electrode polarization and the distributed parameters of the measurement system on these properties, thereby ensuring the acquisition of accurate dielectric characteristics.

### 4.2 Integrating the four-electrode method with the two-electrode method

In pursuit of more precise dielectric properties of biological tissues up to 100 MHz, we have synergized the four-electrode method with the two-electrode method. The four-electrode method was used to measure the impedance below 1 MHz to eliminate the polarization effects of the electrodes and contact resistance. The two-electrode method was used to measure the impedance above 1 MHz to eliminate the effect of stray capacitance and inductance.

To accommodate the measurement demands of four-electrode and two-electrode methods, we have implemented specific enhancements for each. For the four-electrode impedance measurement system, we have integrated the Solartron 1,294 impedance interface, which boasts higher input impedance and greater measurement precision. This addition significantly reduces the effects of electrode polarization, leading to improved accuracy in low-frequency measurements.

For the two-electrode method, we have designed a measurement system with minimal lead length using the Agilent 4294A impedance analyzer, along with the Agilent 42942A terminal adapter and Agilent 16092A clamps. Additionally, we have developed advanced calibration techniques to further mitigate the impact of distributed parameters, thereby enhancing the reliability of high-frequency measurement outcomes.

Based on the analysis of the optimal frequency range (Figure 5), we have proposed recommendations for measuring the dielectric properties of biological tissues across the frequency range from 10 Hz to 100 MHz. The four-electrode method is found to be most effective for frequencies below 50kHz, whereas the two-electrode method demonstrates superior performance at frequencies above 200 kHz. For the intermediate frequencies, an optimal result can be achieved by utilizing a weighted average of the two methods.

The integrated methodology allows us to maintain a low level of measurement deviation across the entire spectrum up to 100 MHz. For NaCl solution, the maximum relative deviation observed is 6.34%, and the average deviation is less than 1.5% (Figure 6; Table 1). For porcine liver tissue, the relative deviation is kept below 6% overall (Figure 7). These improvements are significantly lower than the 10% deviation reported in literature (25), highlighting the superior performance of our method in meeting the stringent demands for measuring biological tissues. This synergy approach not only broadens the frequency range for accurate measurements but also ensures that the results are consistent and reliable across the entire spectrum of frequencies. It provides a robust framework for accurate impedance measurements, particularly in the context of biological applications where precision is paramount.

### 4.3 Preserving the vitality of biological tissues

As documented in various studies, measurement conditions such as temperature, humidity, and the duration that tissue is kept *ex vivo* can significantly influence the measurement results of dielectric property of biological tissues (Di Meo et al., 2022; Fallahi, Sebek, and Prakash, 2021; Halonen et al., 2022; Wang et al., 2023). In this study, we have adopted the following measures to make the environment of the tissue samples as similar as possible to their *in vivo* conditions, thereby ensuring that the dielectric properties of the *ex vivo* tissues closely approximate those *in vivo*.

The samples are maintained in incubator with the same temperature and humidity as *in vivo*. The samples are immersed in physiological saline to replicate the *in vivo* interstitial and tissue fluid environment. All sample measurements were completed within 30 min after clamping the hepatic portal vessels and stopping blood supply to the liver. The short period of tissue ischemia ensures that the tissue remains in an active state similar to that *in vivo* (Wang et al., 2021).

As we look to the future, the implementation of automated sampling and specimen preparation has the potential to significantly reduce the duration of *ex vivo* measurements. This innovation not only streamlines the process but also promises to bridge the gap between *ex vivo* and *in vivo* data, enhancing the correlation between the two. Such progress is expected to yield more reliable and accurate measurement outcomes, thereby reinforcing the significance of the measuring cell as an indispensable tool in biological tissue analysis. Furthermore, by integrating the dielectric property measurement techniques with magnetic property techniques, a more comprehensive understanding of the biophysical characteristics of diseases can be achieved, which in turn provides more accurate guidance for clinical diagnosis and treatment (O'Callaghan et al., 2017; Winkler R et al., 2023).

In the future, we will develop the automated sampling and specimen preparation technologies to reduce the duration of *ex vivo* measurements. Concurrently, we will focus on refining our calibration methods to further minimize system measurement errors. Furthermore, we recognize the importance of considering the sensitivity of the measurement method. This involves not only assessing the method’s reproducibility but also its ability to detect subtle changes in dielectric properties. These efforts will significantly enhance the reliability of the measurement system and methods employed in this study, paving the way for more advanced and reliable dielectric property measurement of active biological tissues across the 10 Hz to 100 MHz frequency range.

In summary, this study has introduced an innovative measurement technique for determining the dielectric properties of active biological tissues within the frequency range of 10Hz to 100MHz, leveraging a newly developed two/four-electrode dual-purpose measuring cell. The efficacy of this method was confirmed by assessing the dielectric properties of NaCl solutions with varying concentrations and porcine liver tissues. The findings highlight the method’s aptness for quantifying dielectric properties in active biological tissues and underscore its potential to serve as a solid foundation for future research in this critical field.

## Data Availability

The raw data supporting the conclusions of this article will be made available by the authors, without undue reservation.
